# Supplementary material for: Mortality Levels and Production Indicators for Suspicion of Highly Pathogenic Avian Influenza Virus Infection in Commercially Farmed Ducks
Source: Pathogens. 2021 Nov 17;10(11):1498. doi: 10.3390/pathogens10111498 (PMC8620262; doi:10.3390/pathogens10111498)
Supplement: Supplementary file 1 [file pathogens-10-01498-s001.zip › pathogens-1425075-supplementary.pdf]

Supplemental information

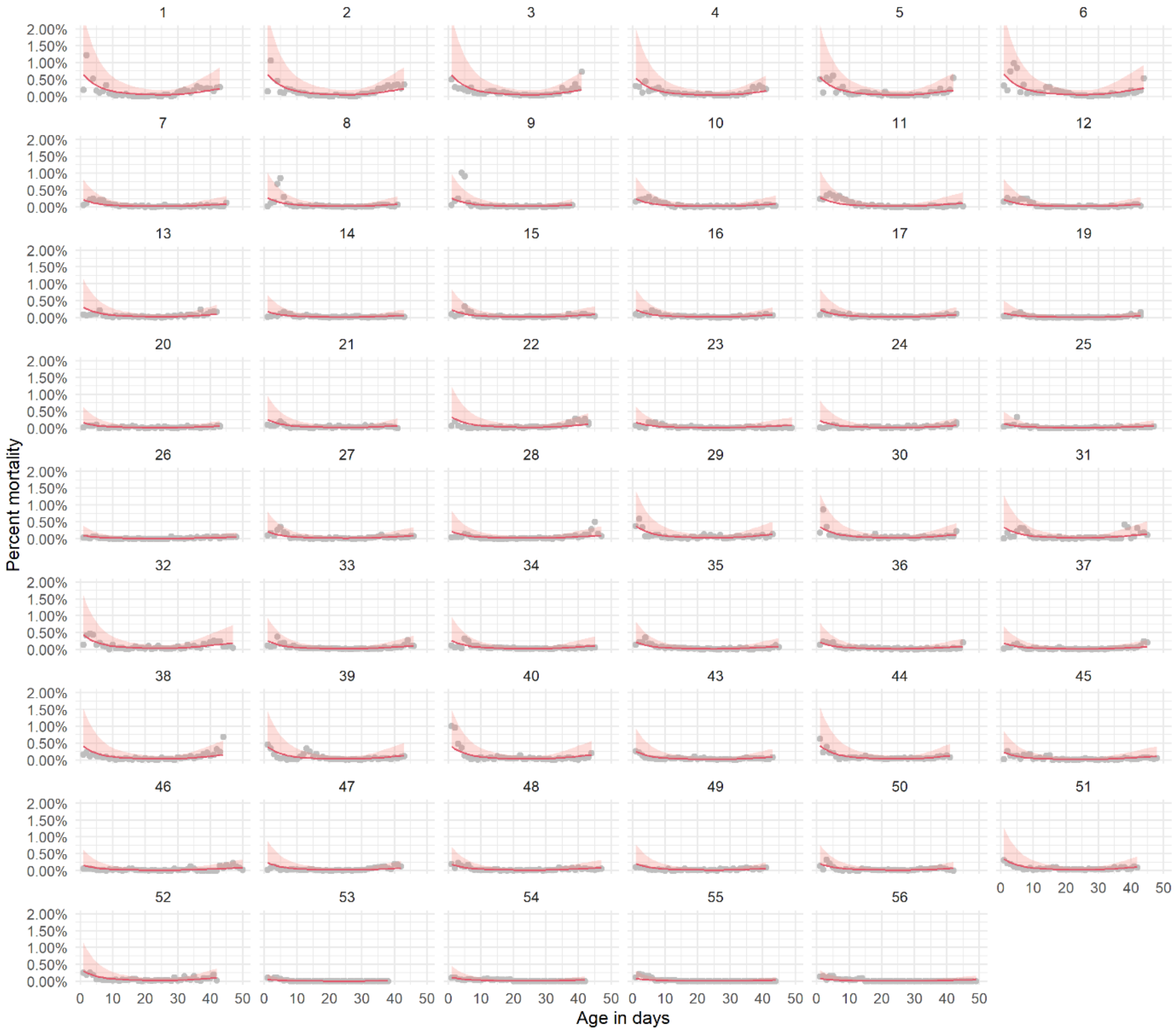

Figure S1. Generalized linear mixed model's results showing the estimated expected daily mortality and 95% upper confidence limit.

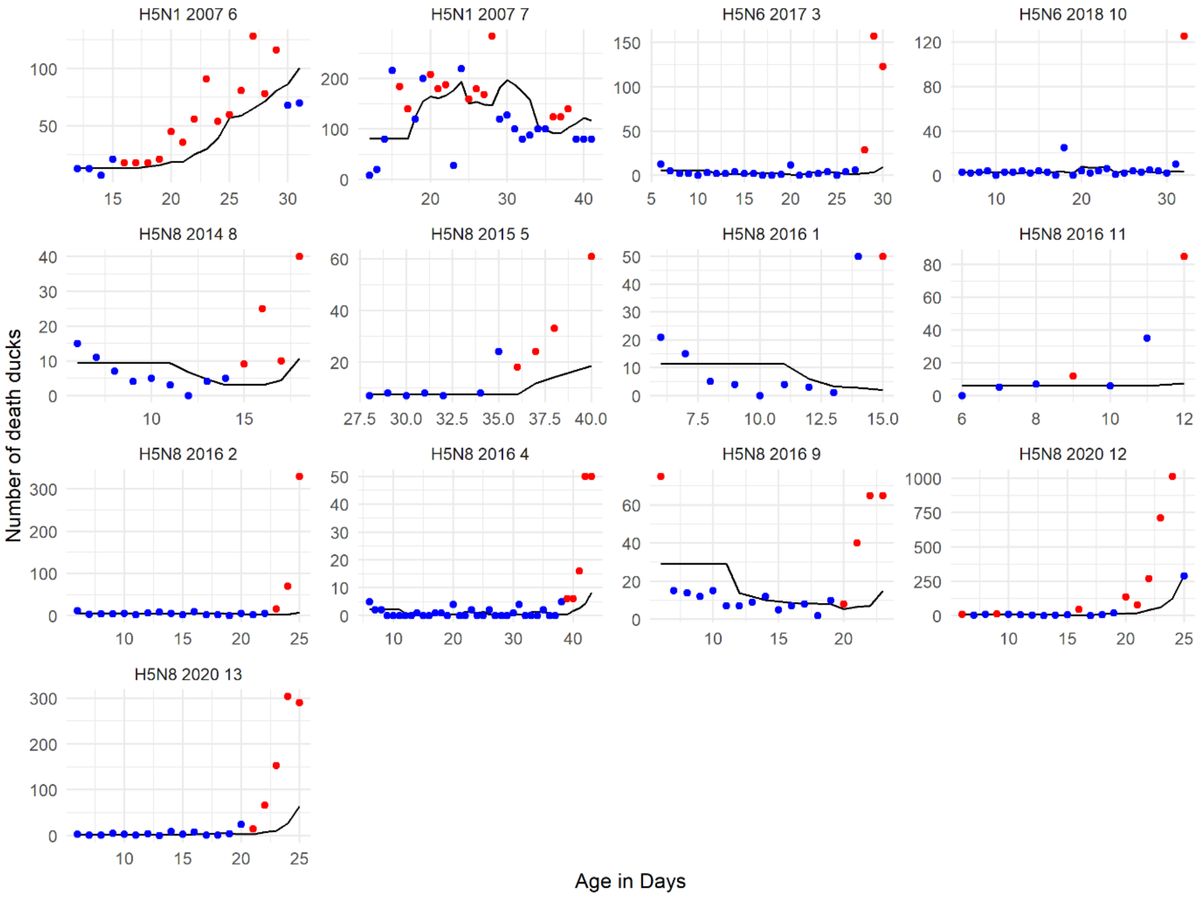

Figure S2 Application of a CUSUM method (EARS-2) for detection of alarms in AI-Infected broiler duck flocks. Red dots indicate raised alarms when thresholds are passed for two consecutive days. This figure gives a graphical overview of the Sensitivity of this method.

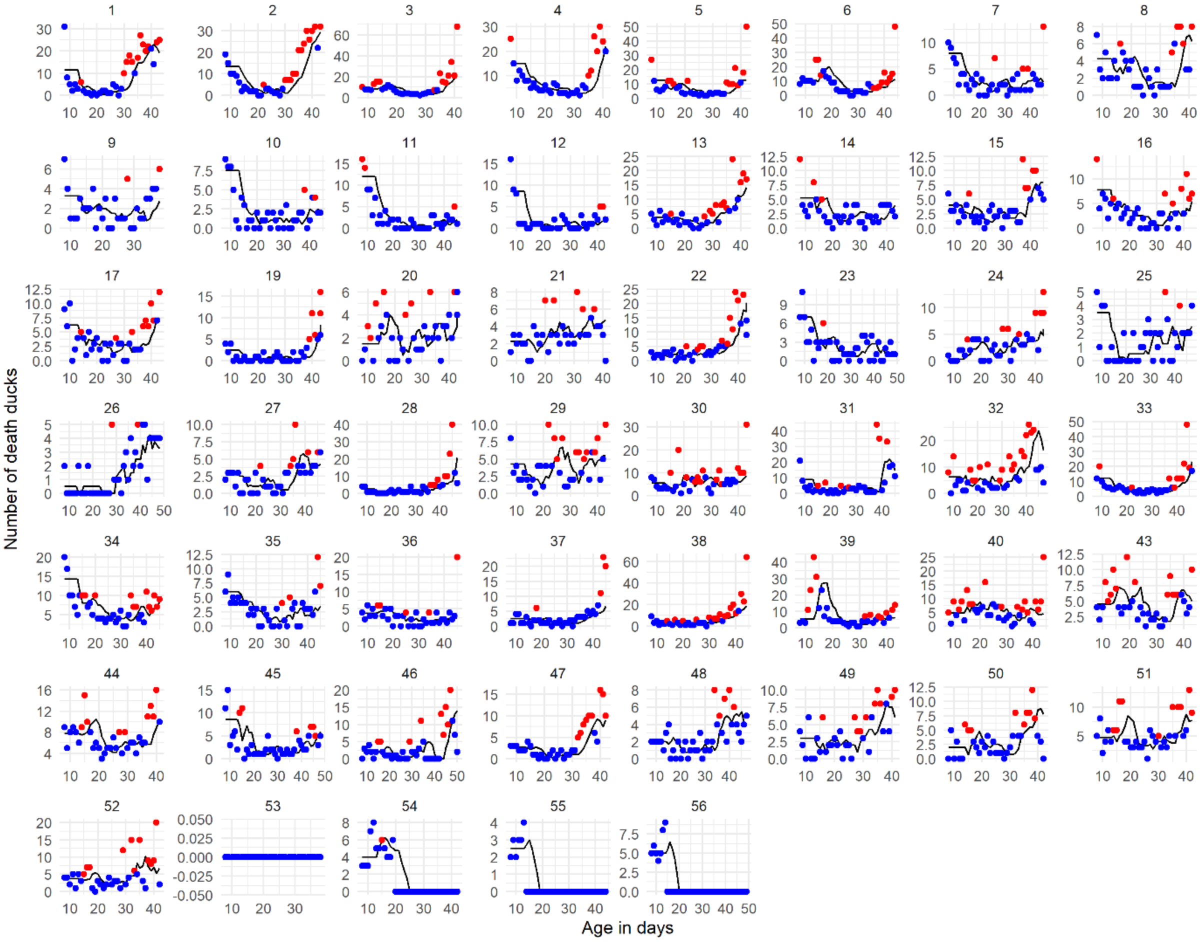

Figure S3. Application of a CUSUM (EARS-2) methods to Non-AI-Infected flocks. All red dots are false alarms raised by the application of these methods. This figure gives a graphical overview of the specificity of this method.

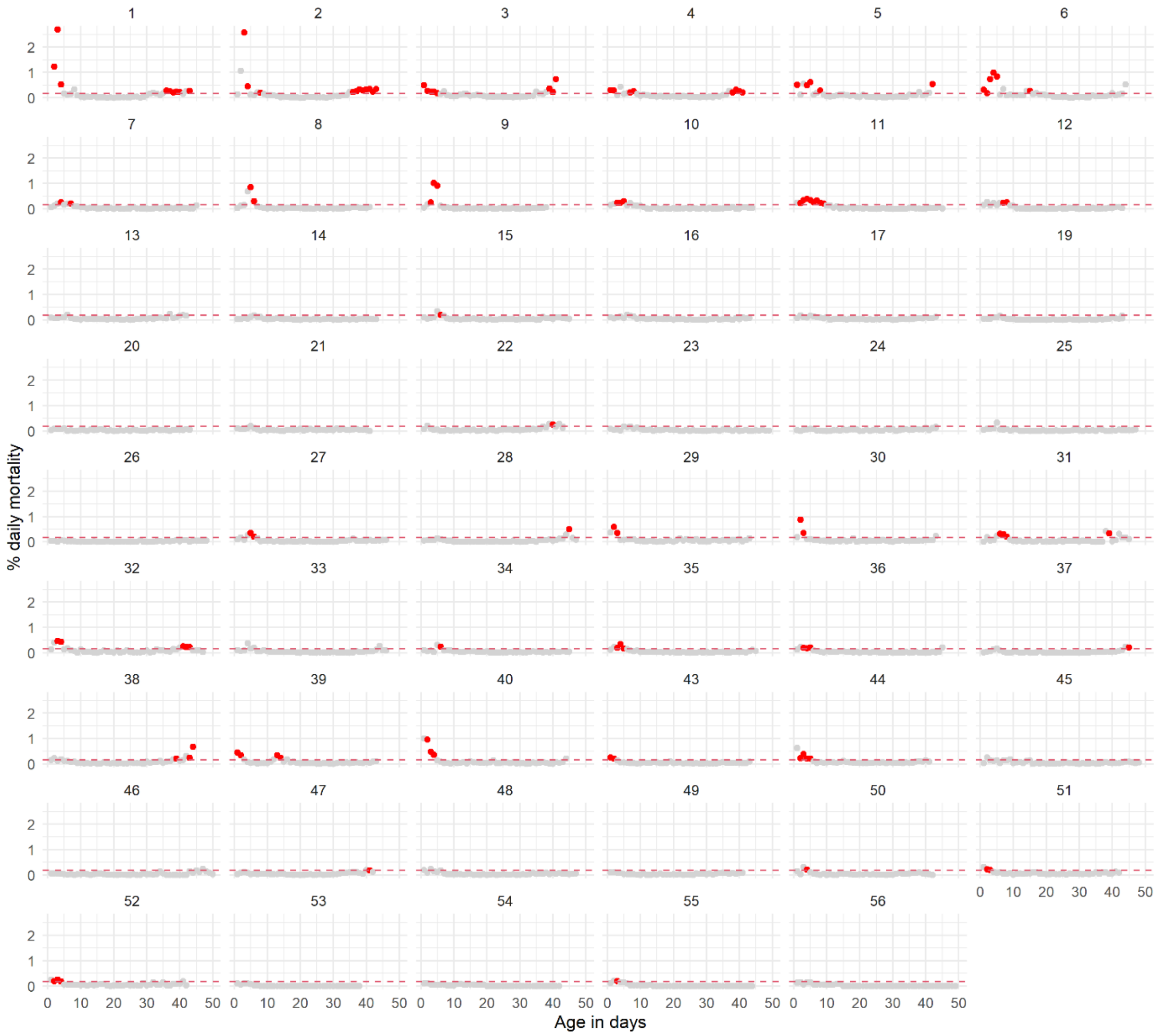

Figure S4. Application of mortality = 0.17% as fixed threshold for raising alarms for HPAI. All red dots are false alarms raised by the application of this method. This figure gives a graphical overview of the specificity of this method.

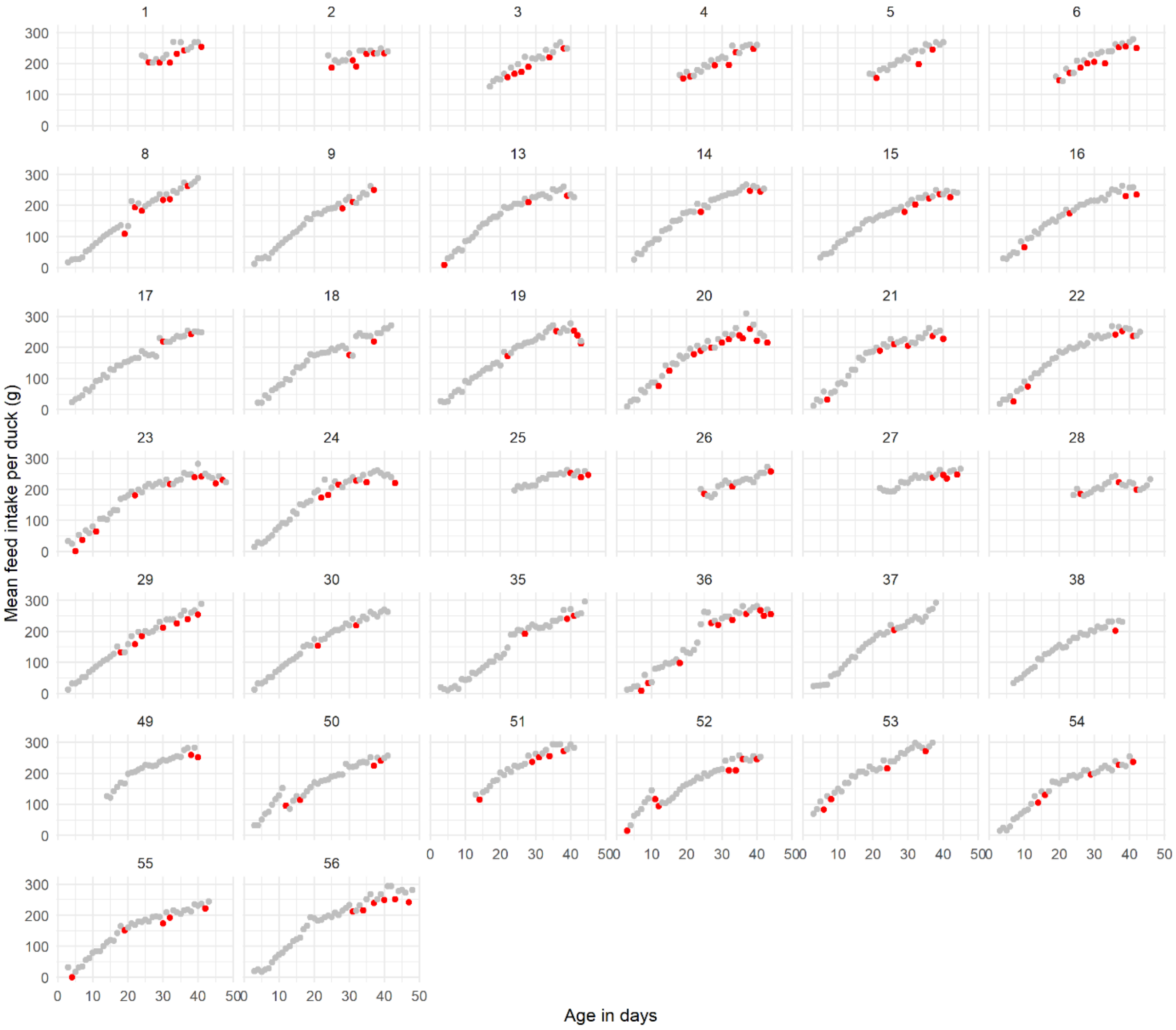

Figure S5. Application of a drop in mean feed intake ≥ 9 grams/duck/day as fixed threshold for raising alarms for HPAI. All red dots are false alarms raised by the application of this method. This figure gives a graphical overview of the specificity of this method. A threshold ≥ 7 grams/duck/day was also assessed which showed higher number of false alarms.

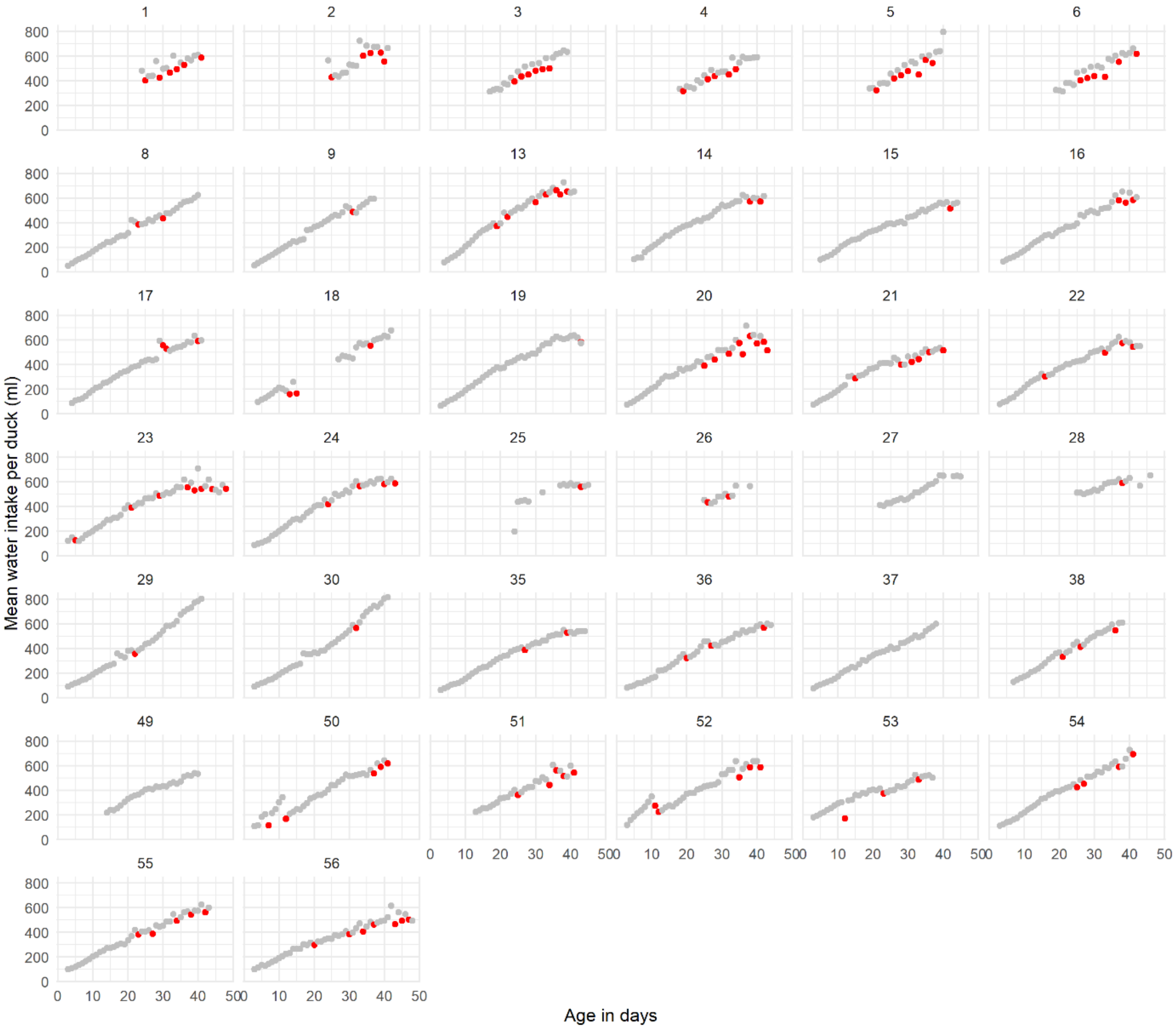

Figure S6. Application of a drop in mean water intake ≥18 ml/duck/day as fixed threshold for raising alarms for HPAI. All red dots are false alarms raised by the application of this method. This figure gives a graphical overview of the specificity of this method. A threshold ≥14 ml/duck/day was also assessed which showed higher number of false alarms.

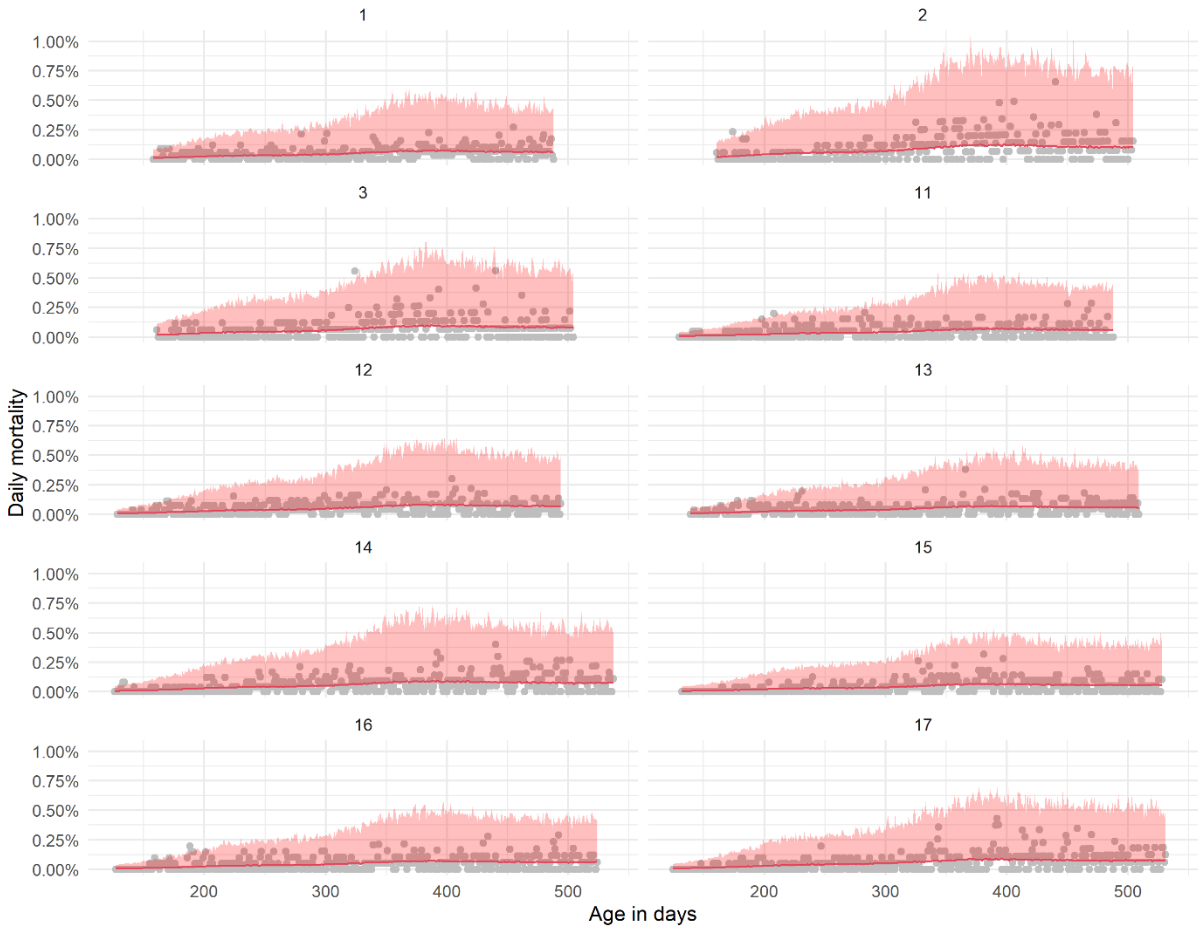

Figure S7. Daily mortality of non-AI-Infected breeder flocks and model estimated expected mortality (red line) and 95% upper confidence limits (red shaded area). One flock had many missing data on daily mortality and was excluded from the analysis.

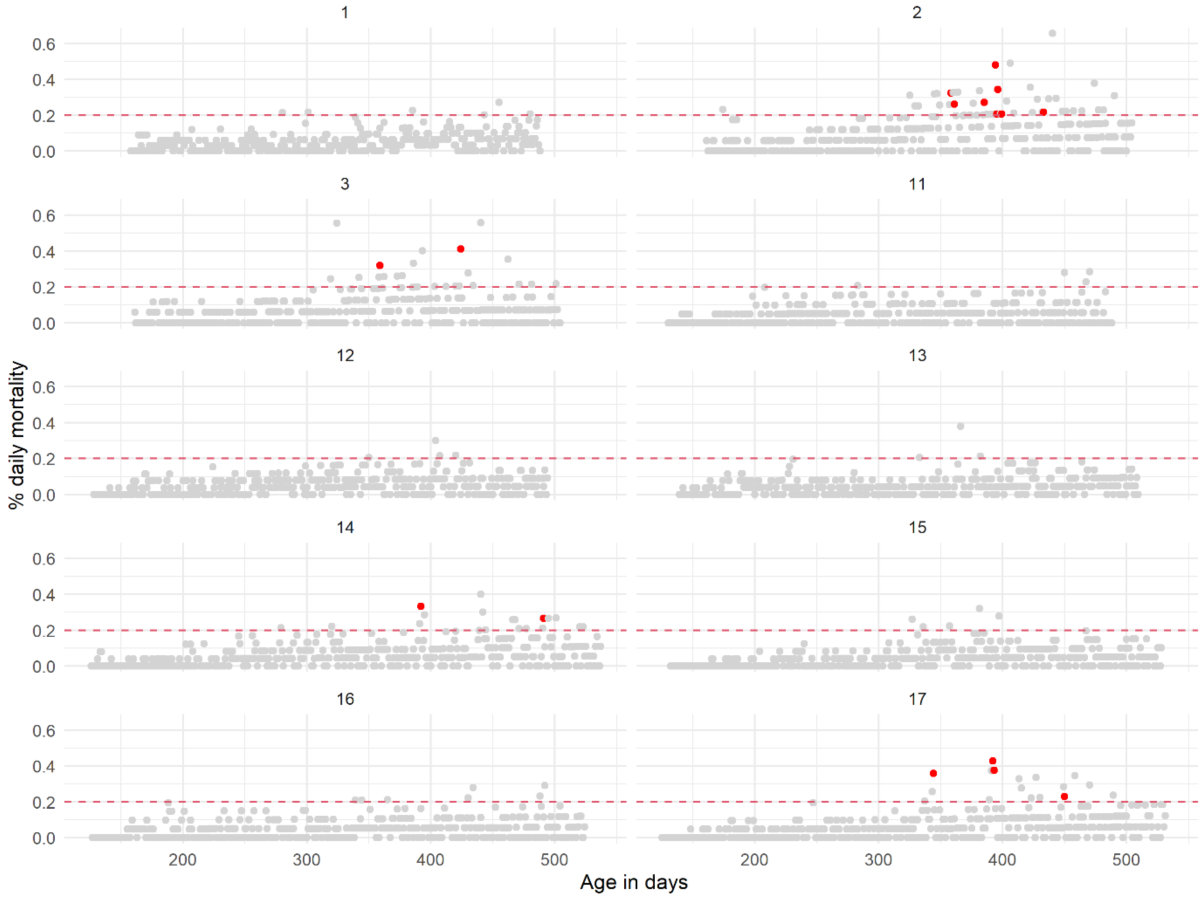

Figure S8. Daily mortality of non-AI-Infected breeder flocks and use of a fixed mortality threshold of 0.2% for raising suspicions of avian influenza infections. Red rods are raised false alarms, following observations in mortalities higher than 0.2% for two consecutive days.

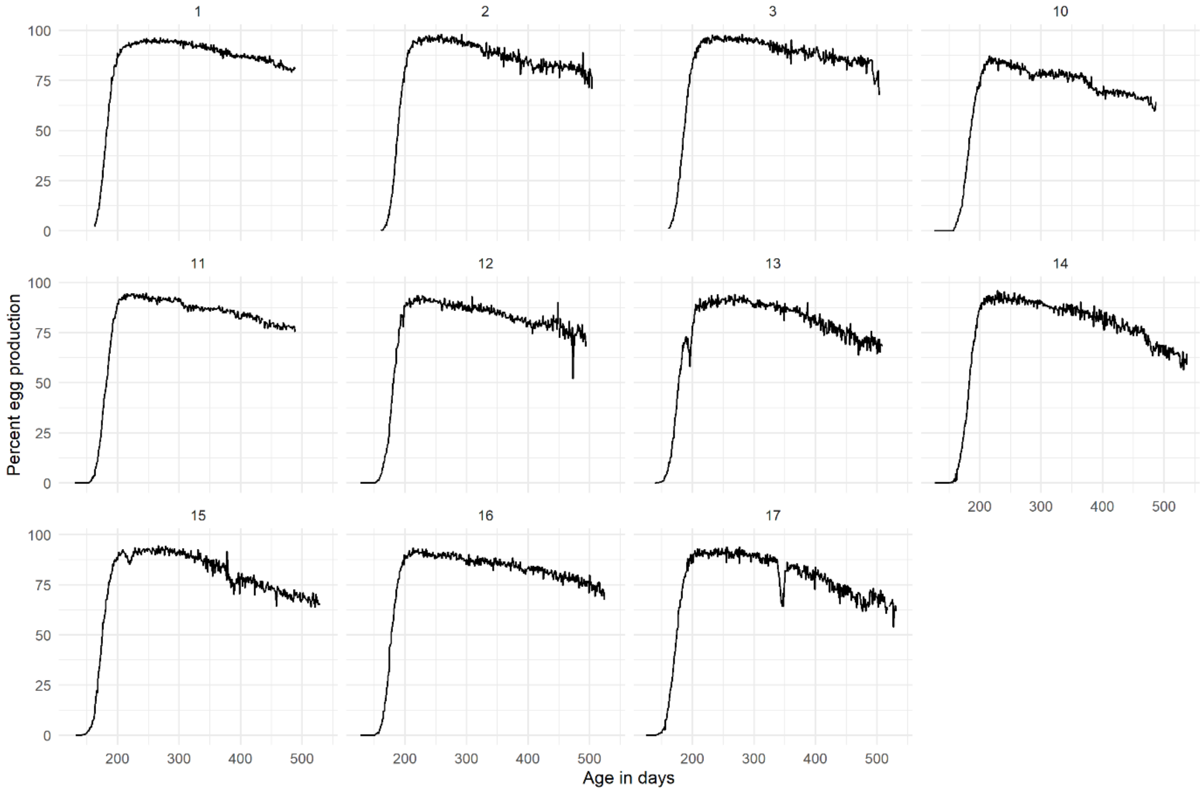

Figure S9. Daily egg production from the 11 non-AI-Infected breeder flocks used for analysis.

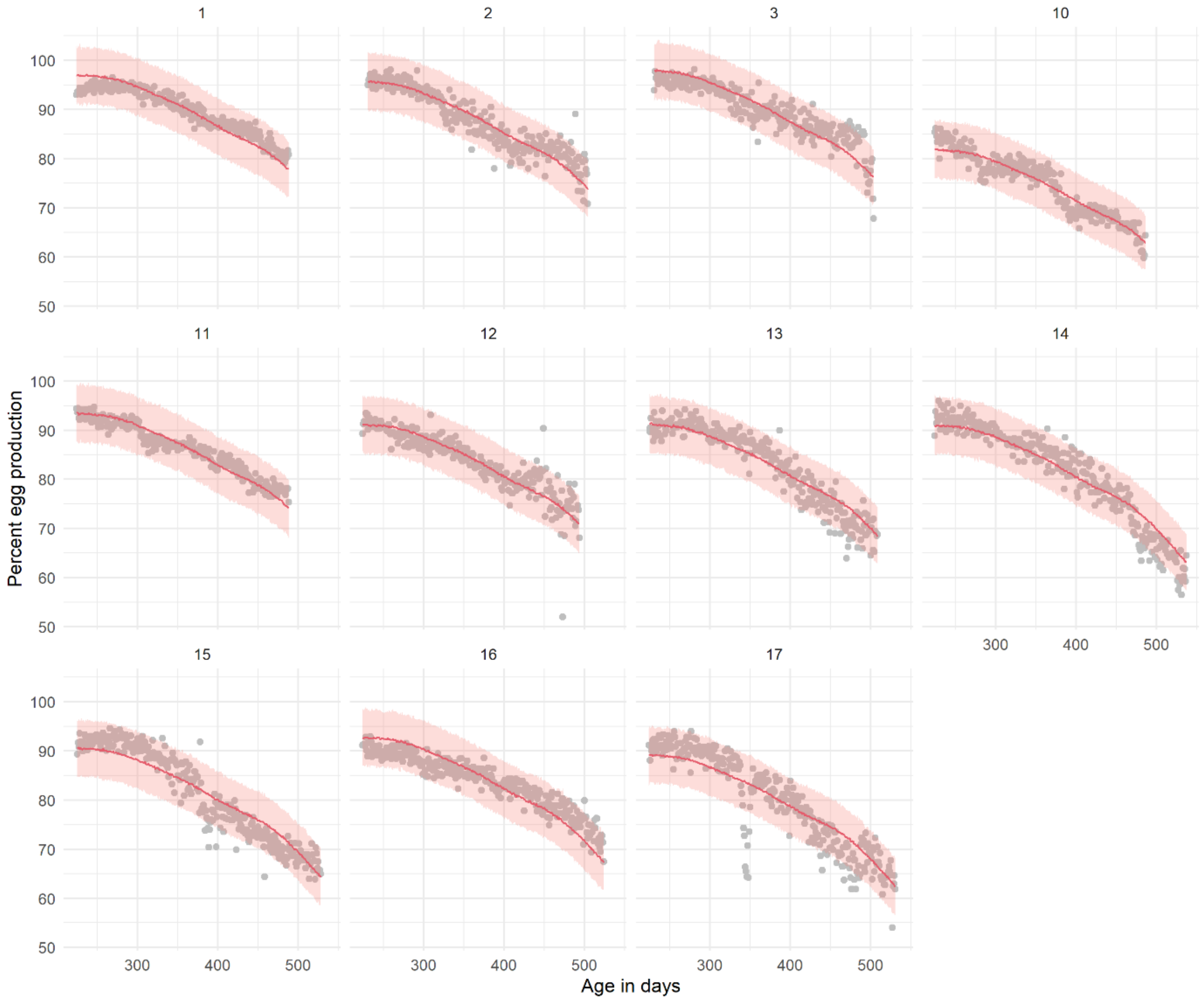

Figure S10. Linear mixed model estimates of mean egg production and 95% Confidence intervals. The model was fitted to data starting on week 32 (252 days) of age.

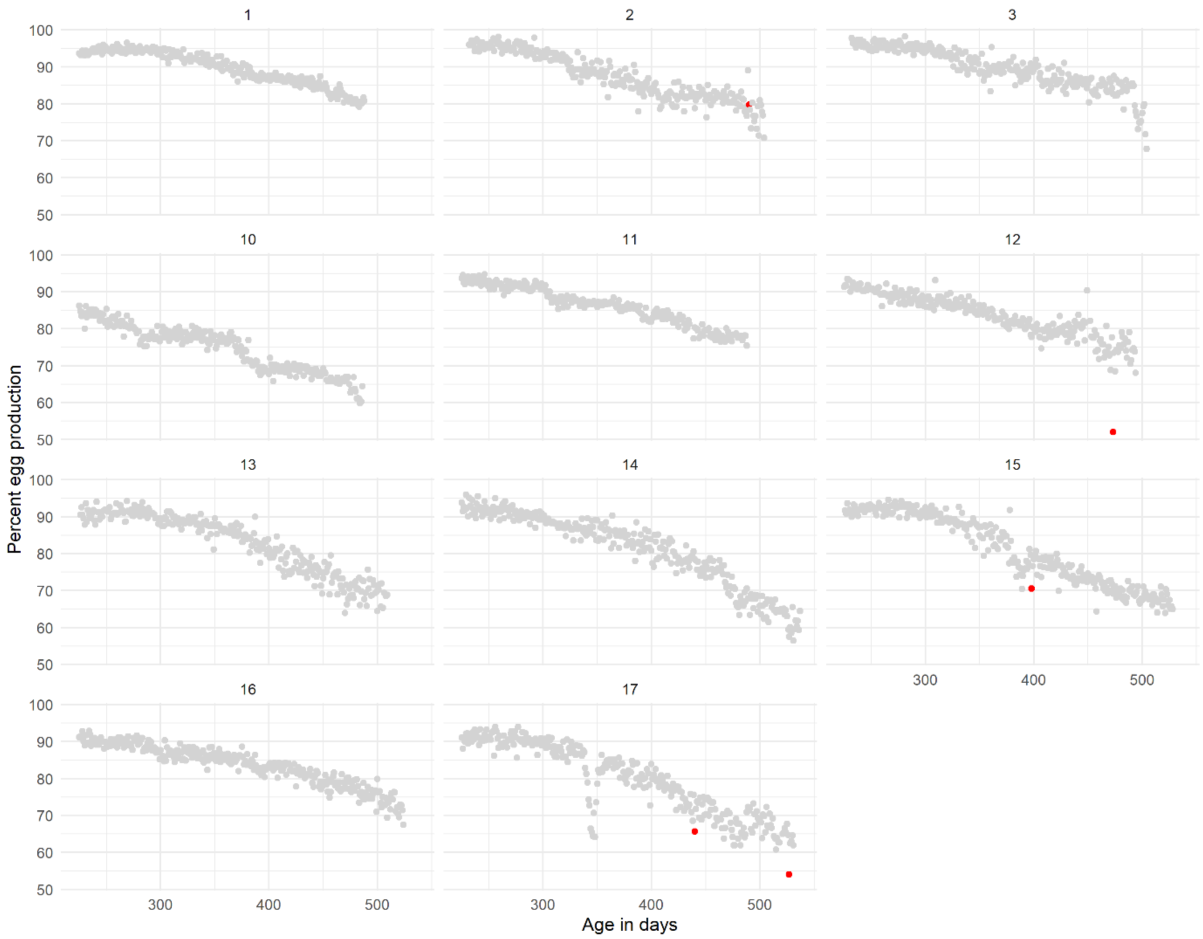

Figure S11. Identifying days when the percent drop in egg production is larger than 9% in relation to the previous day in non-AI-Infected breeder flocks.

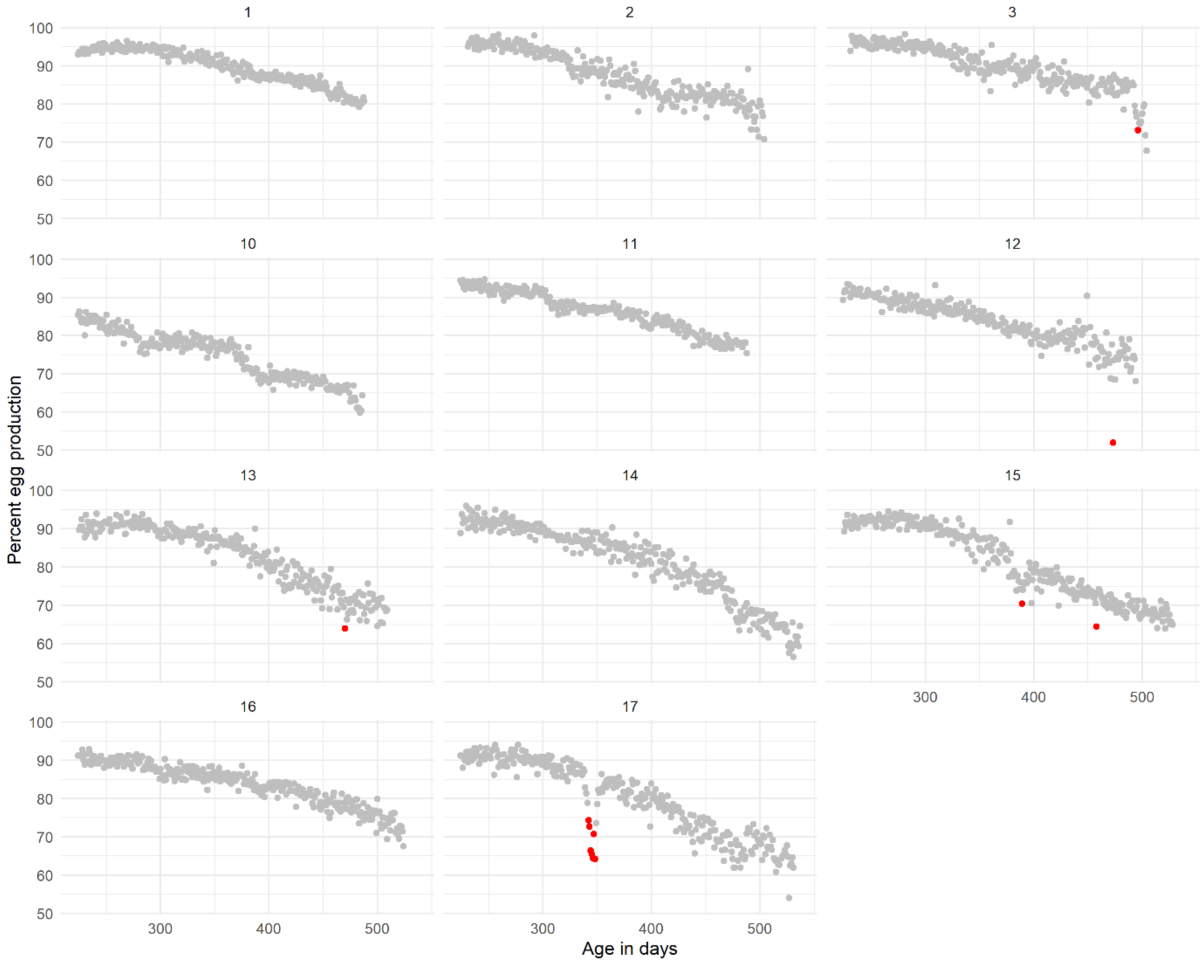

Figure S12. Identifying days when the percent drop in mean egg production is larger than 9% in relation to the mean production the previous week in non-AI-Infected breeder flocks.

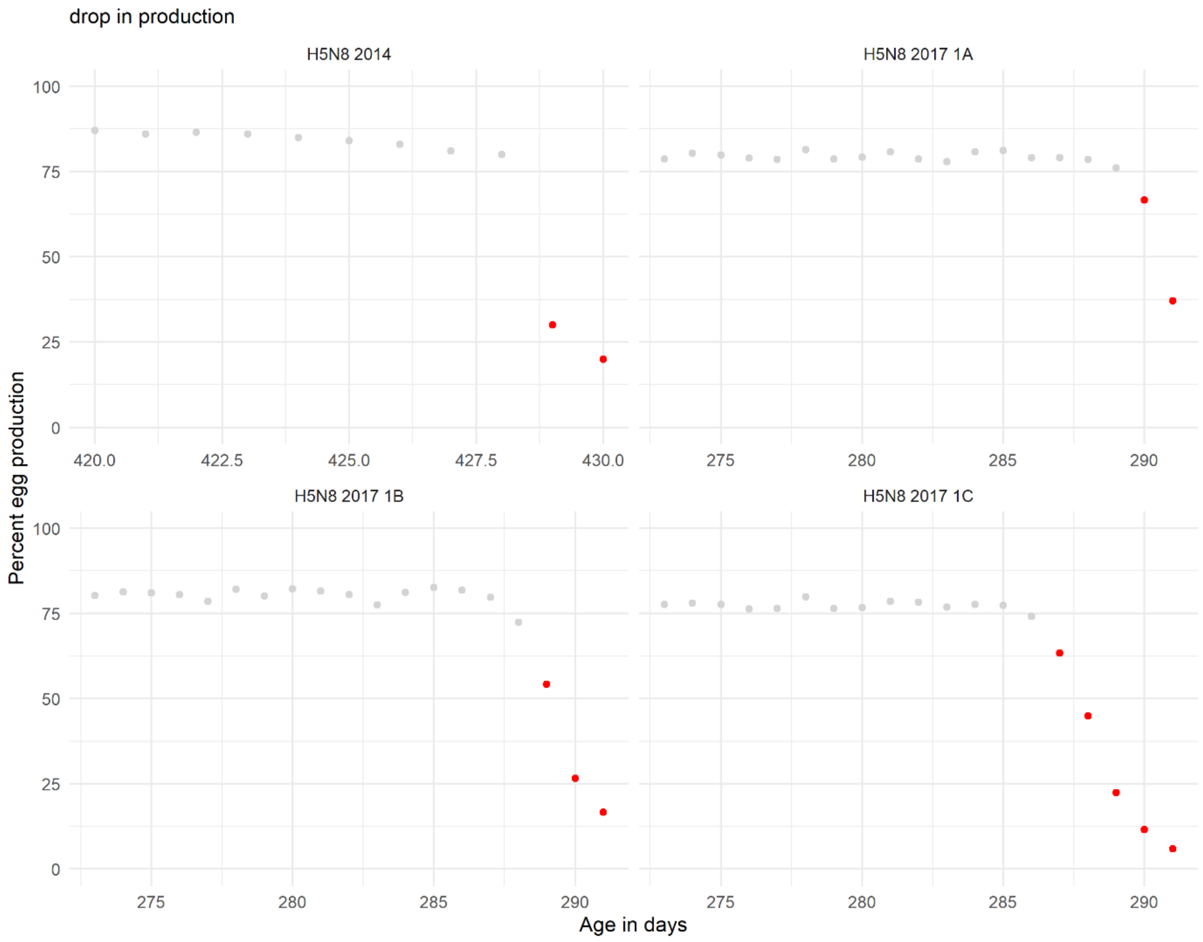

Figure S13. Daily egg production data from breeder flocks affected with HPAI. The virus subtype and year of the outbreak are given as titles for each panel. For flocks affected with the same HPAI virus subtype the same year a flock identifier (1A, 1B,1C) is provided. Red dots identify days when the percent drop in production is equal or bigger than 9% of the mean production the previous week.

Table S1. Negative binomial GLMM fitted to model daily mortality in Non-AI-Infected broiler duck flocks. Parameter values are presented as log estimates.
Knots for natural spline (ns) effects with 5 degrees of freedom (ns(age_days, df = 5))

Knot number	Age in days
1	9
2	18
3	27
4	36

Variable	estimate	std.error	statistic	p.value
Intercept	-6.004	0.101	-59.213	0.000
ns 1	-2.293	0.096	-23.922	0.000
ns 2	-2.934	0.122	-24.092	0.000
ns 3	-0.823	0.106	-7.781	0.000
ns 4	-2.124	0.196	-10.849	0.000
ns 5	0.146	0.157	0.933	0.351
Flock id standard deviation		0.546		
Residual standard deviation		0.700		
Number of flocks		53		
Conditional R^2		0.63		

Table S2. Poisson GLMM fitted to model daily mortality in breeder flocks during their production period
Knots for natural spline (ns) effects with 5 degrees of freedom (ns(age_days, df = 5))

Knot number	Age in days
1	213
2	287
3	362
4	437

Variable	estimate	std.error	statistic	p.value
Intercept	-9.726	0.181	-53.781	0.000
ns 1	1.775	0.161	11.011	0.000
ns 2	2.758	0.194	14.195	0.000
ns 3	1.814	0.120	15.134	0.000
ns 4	4.121	0.382	10.776	0.000
ns 5	1.376	0.112	12.272	0.000
Flock Id standard deviation		0.191		
Residual standard deviation		0.812		
Number of flocks		10		
Conditional R^2		0.4		

Table S3. Linear mixed model fitted to model egg production in non-infected breeder flocks.

Model is fitted starting when breeders were close to peak of production (age 225 days) to the end of production.

Knots for natural spline (ns) effects with 6 degrees of freedom (ns(age_days, df = 6))

Knot number	Age in days
1	272
2	320
3	367
4	414
5	462

Variable	estimate	std.error	statistic	df	p.value
Intercept	92.00	1.35	68.28	10.63	0.00
ns 1	-3.85	0.29	-13.26	3102.00	0.00
ns 2	-7.06	0.37	-18.96	3102.01	0.00
ns 3	-12.19	0.32	-37.90	3102.01	0.00
ns 4	-15.36	0.29	-52.21	3102.06	0.00
ns 5	-23.51	0.62	-38.03	3102.04	0.00
ns 6	-27.68	0.39	-70.20	3102.36	0.00
Flock id standard deviation		4.40			
Residual standard deviation		2.63			
Number of Flocks		11			
Conditional R^2		0.9			

Table S4. Generalized mixed model (binomial) fitted to the daily probability of egg production during the drop in production period (4 to 6 days) before detection.

Variable	Log Odds	std.error	statistic	p.value	conf.low	conf.high
(Intercept)	2.238	0.271	8.267	0.000	1.708	2.769
days	-1.004	0.012	-83.532	0.000	-1.028	-0.981
Flock id standard deviation		0.538				
Residual standard deviation		1.810				
Number of Flocks		4				
Conditional R^2		0.4				
